# Peer services in behavioral health: A scoping review of Medicaid funding to inform policy and practice for refugee and newcomer populations in the U.S.

**DOI:** 10.1371/journal.pmen.0000359

**Published:** 2025-07-10

**Authors:** Mary Bunn, McKenzie Graunke, Xia Liu, Nihmot Abebayo, Sara Izquierdo, Charlene Sunkel, Judith A. Cook

**Affiliations:** 1 Department of Psychiatry, University of Illinois Chicago, Chicago, Illinois, United States of America; 2 Center for Global Health, University of Illinois Chicago, Chicago, Illinois, United States of America; 3 College of Medicine, University of Illinois Chicago, Chicago, Illinois, United States of America; 4 Global Mental Health Peer Network, Paarl, South Africa; 5 Center on Mental Health Services Research & Policy, University of Illinois Chicago, Chicago, Illinois, United States of America; PLOS: Public Library of Science, UNITED KINGDOM OF GREAT BRITAIN AND NORTHERN IRELAND

## Abstract

Refugee and other newcomer communities are at high risk for common mental health and psychosocial problems due to violence and trauma experienced in the country of origin and during migration, both of which are compounded by displacement stressors. Despite significant need, there are major gaps in the behavioral health service system available to refugee and newcomer communities in the U.S. Peer-delivered services have a potentially important role to play in promoting the behavioral health of refugee and newcomer communities domestically. To encourage model development and implementation, this scoping review examined Medicaid-financed peer services in the U.S in the fields of adult substance use, adult mental health, and child and adolescent mental health. Ten publications were included in the analysis. Most states provided Medicaid reimbursement for peer services in some fields but not others. The definition, credentialing processes, and reimbursement rates for peer providers varied across states. To better integrate peer services for refugee and newcomer communities, the findings indicate a need to expand the conceptualization of lived experience to include forced displacement, refine training approaches to empower refugee communities, and adapt and test evidence-based peer models that promote mental health during resettlement and integration. Policy attention will be needed to address low Medicaid reimbursement rates and expansion of Medicaid coverage for newcomers, to encourage and sustain growth in peer support services that enhance mental health after forced migration.

## Introduction

As of October 2024, there were more than 122 million individuals forcibly displaced from their homes worldwide due to conflict, persecution, and human rights violations, as well as natural disasters and climate change [1]. These events force individuals to flee their homes and seek safety in other countries. Approximately one third of these individuals are registered as refugees. A refugee is an individual who was persecuted or fears persecution due to race, religion, nationality, political opinion, or membership in a particular social group [2]. Most refugees remain displaced in urban settings and refugee camps in low and middle-income countries (1). A portion of refugees worldwide are resettled to a third country including the U.S. *Third country* refers to a country different from their country of origin or country where they sought asylum (1). While the number of refugees admitted to the U.S. ebbs and flows annually, approximately 3.6 million refugees have been resettled to the U.S since the refugee admissions program was established in 1980 [3]. There are also other newcomer populations in the U.S. who have fled their countries to escape violence and instability. This includes asylum seekers and asylees (Asylum-seekers are individuals who are already in the United States and have filed an application with the U.S. government because they have suffered or will suffer persecution due to race, religion, nationality, membership in a particular social group, or political opinion.) [4] and those with humanitarian parole (Humanitarian parole can be granted to an individual outside of the U.S. to allow them temporary admittance into the U.S. due to urgent humanitarian reasons or significant public benefit.) [5] or temporary protected status (Temporary Protected Status may be designated to people who have come from a particular country due to conditions that temporarily prevent the country’s nationals from returning safely due to ongoing armed conflict, environmental disaster, or other extraordinary conditions.) [6]. In this paper, we use *refugee and newcomer* to refer to populations that have recently arrived to the U.S.

Refugee and other newcomer communities are at increased risk for mental health and psychosocial problems due to violence and trauma experienced in their country of origin and during migration, both of which are compounded by postmigration stressors and living difficulties [7–9]. This includes high rates of depression, anxiety and PTSD [10], substance use, and substance use disorders [11–13] for adult, child and adolescent populations. Forced migration also results in social losses and family-level problems due to family separation, change in family roles, and disruption in community connections [14–17]. For example, recent research conducted with Syrian and Iraqi families resettled in the U.S. identified novel stressors that mothers faced due to the losses of supportive caregiving networks, safety concerns, new norms, environmental changes and new parenting demands [18]. A review of social support research conducted with resettled refugee communities identified multi-dimensional losses to social support [19] that adversely impact relationships within families and break down vital social networks of support and connection [20,21].

In the U.S., the federal Office of Refugee Resettlement and the Centers for Disease Control and Prevention recommend that newcomers receive a behavioral health assessment shortly after arrival that includes screening for depression and PTSD followed by referrals for any needed specialized treatment. However, a survey of state refugee health coordinators in 2015 found persistent shortfalls in the delivery of behavioral health assessments and referrals to these populations – more than half indicated that it was not possible to administer a brief mental health screening given current resources and capacities [22]. Thus, despite significant need, there are major gaps in the behavioral health service system available to refugee and other newcomer communities in the U.S.

There are limited mental health services available to refugee and newcomer communities upon arrival in the U.S [23]. Only a few organizations specialize in delivery of refugee mental health services and those that are provided often focus narrowly on specialized clinical care and individual psychotherapies [24]. While access to specialized clinical care is important, addressing large-scale mental health needs requires a broader approach emphasizing community-based and community driven psychosocial models that respond to the wide-ranging impacts of trauma and chronic adversity on individuals, families, and communities and which empower local communities to deliver needed services and care [14].

Peer-delivered services have a potentially important role to play in this broader service paradigm. A peer is a person with shared life experiences with mental health conditions and recovery who draws upon these experiences along with specific training to assist others to enhance their mental health [25–29]. Systematic reviews in the areas of child mental health, adult mental health, and substance use services indicate that peers can effectively deliver a range of mental health and behavioral health prevention and support interventions [30–32]. The shared experience between peer and service user is associated with an enhanced working alliance, reductions in stigma about mental health services, enhanced social support, and engagement in services [33] and research has demonstrated positive psychosocial and clinical outcomes [34,35].

In global mental health and humanitarian emergency settings, a well-known strategy to address mental health needs in contexts with limited formal resources is called “task shifting” or “task sharing.” This involves assigning or shifting mental health activities typically performed by more specialized health workers to those who are less specialized such as community health workers or peers along with appropriate training and supervision. Research from global settings indicates that task sharing models have been used to effectively deliver a range of behavioral health interventions such as individual and group psychotherapies [36], maternal mental health interventions [37], psychosocial services, [38] and mental health promotion interventions [39], with mixed though mainly favorable results for alleviating symptoms of depression, anxiety, PTSD, and substance use [40]. Successful use of peers to provide support to refugee communities outside the U.S. suggests that such services are associated with improved emotional health and lower psychological stress [41] as well as benefits at the interpersonal, organizational and community levels. For example, a qualitative study of a peer support group for Arabic-speaking refugees in Canada found that participants reported individual level benefits such as increased sociability and resilience, improvements in family functioning, improved self-efficacy in accessing community services and an increased interest in participating in community development activities [42]. A study in Spain found significant improvements in appreciation of life, personal strength, and relating to others following a 15-week peer mentoring program for refugee communities [43].

### Peer services and Medicaid

Despite promising findings globally, with some notable exceptions, there has been limited implementation of task sharing models using peers to promote the behavioral health of refugee and newcomer communities in the U.S. [44]. Medicaid is the primary funder of peer support services in the U.S., following the release of a 2007 directive from the Centers for Medicare and Medicaid Services (CMS) allowing peer services as a reimbursable service so long as they are supervised by a mental health professional [45]. This is relevant to newcomer communities since over 60 percent of those age 16 + have public insurance in the form of Refugee Medical Assistance (RMA), ORR-funded Medicaid coverage, the Children’s Health Insurance program (CHIP), or other sources of public health insurance [46,47]. Policy focused research is needed to understand current Medicaid funding and reimbursement policies and how they can support long-term economic viability of peer services to newcomer communities in the U.S. This research can encourage model development and opportunities for implementation of peer services.

Existing reviews of Medicaid coverage of peer services examine specific fields of peer services (i.e., substance use or severe mental illness) or specific aspects of peer services (e.g., credentials and reimbursement codes). To inform and promote expansion of peer services to refugee and newcomer populations, a comprehensive understanding is needed of how different states define peers, credentialing requirements, and procedures for payment of services across the fields of adult substance use and adult mental health, and child and adolescent mental health. The purpose of this scoping review, therefore, was to conduct a comprehensive comparative analysis of different U.S. states’ Medicaid policies and practices covering the delivery of peer support services for behavioral health. In doing so, we intended to summarize current Medicaid practices to highlight opportunities to strengthen integration of peer services for refugee and newcomer communities and use Medicaid dollars to sustain funding for these programs.

## Methods

The study is informed by a peer support theoretical framework which posits that recovery from mental health conditions and related challenges is promoted through a symmetrical relationship between service recipients and peers who have shared life experiences including shared experiences of mental health challenges and use their skills and experiences to aid others. This relationship involves shared histories, unconditional acceptance, empathy, and a belief in recovery, offering opportunities for role modeling and developing self-management skills, leading to enhanced self-esteem and hopefulness [48–51].

The research team included both peer and non-peer researchers. This allowed for multiple perspectives regarding the role of lived experience in service delivery, peer certification, and funding for peer services and their expanded use in services for refugees. The team also included individuals with diverse expertise including individual and family histories of migration, and use of peers to provide services and supports in programs for refugee communities.

We used a scoping review methodology to identify and map the evidence on Medicaid practices and policies related to funding peer support services in behavioral health [52]. We used this methodology because our aim was identify and analyze literature on a predetermined topic by addressing specific questions, which is the primary purpose of scoping or mapping reviews [53]. In contrast, systematic reviews are designed to answer a narrower question of effectiveness and therefore were not well-suited for our study’s purposes [54]. We followed Arksey and O’Malley’s [55] framework for scoping reviews, which includes the following steps: (a) a priori research questions; (b) detailed inclusion and exclusion criteria (c) identifying relevant studies; (d) selecting eligible studies; (e) extracting data and (f) summarizing and synthesizing findings. A protocol was developed to guide the overall review process from inception to completion.

Specific to Medicaid-financed peer services, our review was guided by the following research questions:

1) What are the definitions and qualifications of peer providers utilized?
2) What are requirements for training and credentialing peers across states?
3) In what capacities and for what types of services are peer providers utilized across service domains?
4) What are current Medicaid funding and billing practices for peer support services across states?

### Search strategy

Following Cochrane scoping review guidelines [56], we searched the following databases: PubMed (includes MEDLINE), APA PsycINFO (ProQuest), PAIS Index (ProQuest), Web of Science. These were selected for their extensive coverage of the research topic in consultation with a research librarian specializing in evidence synthesis methods. The involvement of librarians who are trained in advanced search methods and review methodology improves the quality of evidence syntheses [57,58] and has increased dramatically in scoping review projects in recent years [59].

We developed a search strategy around the three search terms of: (1) peer/peers/peer-to-peer, (2) Medicaid, and (3) not peer-reviewed, deriving terms from the existing literature and collective knowledge of the authors. Both controlled vocabulary terms and keywords were used to describe each of these domains (see S1 Appendix for search terms).

### Inclusion and exclusion criteria

Studies focused on Medicaid-financed peer support services were included. We included publications from peer-reviewed journals as well as grey literature such as policy briefs, working papers, dissertations, and reports from government or other organizations produced outside of traditional commercial publishers; this can facilitate discovery of the full body of evidence and provide a more comprehensive representation of the topic [60].

We included publications that: a) examined Medicaid funding for peer support services for behavioral health; b) investigated peer services delivered in one of three domains of adult mental health, adult substance use, and child and adolescent mental health; c) and were published within the last five years. Studies were excluded if: a) they did not include details on Medicaid financed peer services; b) were not specific to behavioral health in the aforementioned domains; or c) were published more than 5 years ago. Medicaid policies are constantly evolving, so a timeline of five years was established to provide a contemporary picture of the policy landscape to guide future implementation and peer programming. Additionally, we chose a five-year time frame because that is the typical length of Medicaid waivers which expand eligibility or benefits beyond the state plan criteria. A state’s Medicaid state plan outlines all of the standard services that are covered under its Medicaid program. Waivers (including the 1915(b), 1915(c), and 1115) allow states to provide other services not included in the state plan, such as services that also benefit non-recipients of Medicaid, services that are administered at home or in the community in lieu of in a facility, or time-limited experimental programs [61]. We anticipated that Medicaid waivers may play an important role in funding and reimbursement for behavioral health services. The five-year time frame also allowed us to pick up any other changes such as Medicaid state plan amendments that may have occurred pre- and post-COVID, allowing us to see whether such changes were temporary or longer-term. Database searches were performed in April 2023 and October 2024, as depicted in the following PRISMA diagram. Because we anticipated that many sources would be from the grey literature, we also conducted a supplemental search in Google using search terms of “peer” and “Medicaid.” These searches returned any pieces of literature that included both terms, which we then reviewed for relevance (See S1 Appendix).

### Data analysis

A standardized data extraction tool was developed by the lead author to capture information on each publication’s characteristics (aims, design, population, service focus and states reviewed), funding information (states, Medicaid funding sources, billing codes) and Medicaid covered peer service types. Characteristics pertaining to peer providers were also tracked including definitions, eligibility, and credentialing. The tool was pilot tested with an initial set of publications, refined, and finalized.

Publications were split between the authorship team and data were extracted verbatim. To enhance the rigor of the review, the first author conducted a second review of the abstracts to confirm decisions for retention based on inclusion and exclusion criteria. Any discrepancies between the initial review by the four authors and the second review were discussed and resolved during weekly meetings. To analyze the data, we used tabulation and summative content analysis techniques [62] and to analyze narrative text we employed inductive and conventional content analysis [63]. We conducted descriptive analyses to examine funding and billing code information. Peer provider definitions and credentials in each state were cross-referenced with that state’s Medicaid coverage because some states offered credentials that were not covered by Medicaid. All credentials that were eligible for Medicaid reimbursement according to that state’s Medicaid Plan and Waivers were summed, and this number was used to generate statistics to describe the landscape of funding by credential. To analyze peer service types, we conducted an examination of all available information across publications and inductively categorized them into service categories. No formal appraisal of bias was conducted (as is consistent with scoping reviews methodologies), however, the authors examined each publications methodology to assess methods used, rigor and inclusion of strategies.

## Results

### Search results

**Fig 1:**
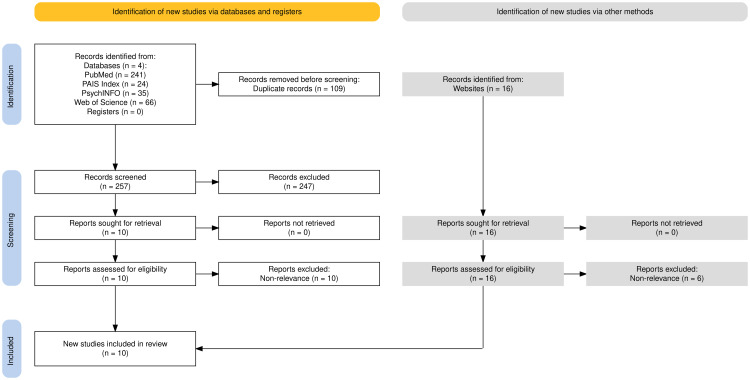
PRISMA flow diagram.

The searches yielded 382 publications in the form of journal articles, reports, data briefs, and summaries: after removing duplicates, the final number of publications was 273. Following the removal of 247 with abstracts that did not meet eligibility criteria, the 26 remaining abstracts were again reviewed by the first author to confirm decisions for retention based on inclusion and exclusion criteria. Most studies were excluded because of non-relevance to Medicaid policies pertaining to peer support. The research team then reviewed the publications’ contents for inclusion, followed by a second review by the first author to ensure the systematic application of inclusion criteria. This led to the exclusion of 16 of the publications due to non-relevance to the context, population or focus resulting in a final sample of 10 review articles (See Fig 1) [64–73].

Table 1 summarizes the ten publications included in the final analysis. All were publicly available. Five were prepared by health research organizations, three by advocacy organizations, one by a policy research organization, and one by a federal government agency. Seven publications focused on peer services for adult mental health and substance use, two on peer services for adults with substance use problems only, and one on child and adolescent mental health delivered by family and youth peer providers. Eight of the ten publications provided details on the methodology used to gather the information presented. All but one included a state-by-state breakdown of peer service provider information.

**Table 1:** Overview of Included Reviews.

Author	Year	Title	Study Aims	Definition of Peer Provider	Fields
Badr	2024	Peer Support Specialists	To provide a concise report offering insight into the emerging issue of peer support specialist around the country.	The Substance Abuse and Mental Health Services Administration defines a peer support specialist (also called a “peer provider”) as someone who “uses his or her lived experience of recovery from mental illness and/or addiction, plus skills learned in formal training, to deliver services in behavioral health settings to promote mind-body recovery and resiliency.”	Adult substance use, adult mental health
Beck	2018	Scopes of practice and reimbursement patterns of addiction counselors, community health workers, and peer recovery specialists in the behavioral health workforce	Analyze data about state Medicaid reimbursement rates, professional training credentials, and scopes of practice for community health workers, peer recovery specialists, and addiction counselors in behavioral health service delivery.	*No definition provided*	Adult substance use
Guth	2023	Medicaid coverage of behavioral health services in 2022: Findings from a survey of state Medicaid programs	Understand variations in access to behavioral health services for adults in Medicaid by surveying behavioral health benefits covered for adult enrollees in their fee-for-service programs.	Individuals who have personally experienced behavioral health challenges.	Adult mental health, Adult substance use
Mette	2019	50-State Scan: How Medicaid agencies leverage their non-licensed substance use disorder workforce	Conduct a comprehensive review of how each state Medicaid program pays for and oversees non-licensed SUD staff and summarize what steps states are taking to reduce workforce shortages.	Individuals with lived experience providing Medicaid-billable substance use disorder services.	Adult substance use
Open Minds	2018	State Medicaid reimbursement for peer support services	Examine state Medicaid billing practices related to peer services and describe whether they allow peer support for both addiction and mental health, only mental health, or only addiction.	People who have lived experience and have been trained to assist others in initiating and maintaining long-term recovery and enhancing the quality of life for individuals and their families.	Adult substance use, Adult mental health
Peer Recovery Center of Excellence	2023	Comparative Analysis of State Requirements for Peer Support Specialist Training and Certification in the United States	To provide a comprehensive overview of state peer recovery trainings and certifications, information offered in this report includes peer support training and certification for peer support specialists with lived experience in substance use and/or mental health recovery. This approach also reflects the integrated certifications for peer support specialists with either type of lived experience offered by many states	Person with lived experience in recovery from mental health and substance use challenges who provides non-clinical, strengths-based support to others seeking their own, individualized, person-centered recovery.	Adult substance use, adult mental health
Peer Recovery Center of Excellence	2024	Medicaid Reimbursement for Peer Support Services: A Detailed Analysis of Rates, Processes, and Procedures	This report provides a national overview of: which states offer Medicaid reimbursement specifically for peer support services; reimbursement rates for one-on-one and group peer support services; funding authority types and selections by each state; billing codes and modifiers used by each state to specify peer support services; and recommendations.	*No definition provided*	Adult substance use, adult mental health
Schober and Baxter	2020	Medicaid funding for family and youth peer support programs in the United States	Catalog state’s efforts and identify states that reimburse for peer support services and state information about Medicaid funding mechanism, billing codes, reimbursement rates, and workforce qualifications.	Family and youth peer support workers are integral members of the treatment team who have personal experience and firsthand knowledge of child- and family-serving systems.	Child and adolescent mental health
United States Government Accountability Office	2020	Substance use disorder: Medicaid coverage of peer support services for adults	Describe which state Medicaid programs covered peer support services nationwide, how state Medicaid programs offered peer support services, and the effects of peer support services to beneficiaries’ health and cost of care.	Peer providers share their own lived experience of recovery along with practical guidance to assist others to initiate and maintain recovery and enhance their quality of life.	Adult substance use
Videka	2019	National analysis of peer support providers: Practice setting, requirements, roles, and reimbursement	Use SAMSHA data to define the organizational setting, roles of peer providers, scopes of practice, and types of facilities that are employing peer specialists.	Someone who has experienced the healing process of recovery from psychiatric, traumatic and substance use and as a result, can offer assistance and support to promote another peer’s own personal recovery.	Adult substance use, Adult mental health

### Definition of peer providers

We examined available definitions of peer providers to understand how peers were conceptualized and capture any relevant variation across behavioral health fields. Seven of the ten publications provided definitions of peers. Of these, only one [69] was specific to adult substance use; the six other publications contained definitions that encompassed multiple domains of lived experience (Table 1).

We identified two main elements across available definitions. First, most definitions identified peers as having lived experience of healing or recovery from mental health or addiction-related problems, identified in six of seven definition (85%). Videka et al. (2019), for example, defined peers as, “someone who has experienced the healing process of recovery from psychiatric, traumatic and substance use and as a result, can offer assistance and support to promote another peer’s own personal recovery.” Second, peers were also defined by their ability to draw on their experience of healing or recovery to assist others(n = 5). Badr (2024) defined a peer as one who uses their lived experience of recovery from mental illness and/or addiction to promote mind-body recovery and resiliency. Another definition described peers as individuals who, “share their own lived experience of recovery along with practical guidance to assist others to initiate and maintain recovery and enhance their quality of life” [66]. Less common elements defined peers as people who are trained (n = 2) and have specific knowledge and experience with the service system (n = 1).

### Eligibility and credentialing of peers

The eligibility requirements for peer support credentials differed across states and behavioral health fields (see Table 2). In the context of peer support for adults with substance use, eligibility was consistently dependent on lived experience with recovery from a substance use disorder (by 37 states, or 74% of states offering a peer adult substance use credential). Six states (12%) required that peers’ recovery experiences be abstinence specific. Only three states did not specify that peer supporters must have specific personal experiences with substance use [73]. Peers in the domain of adult mental health were required to have personal experience recovering from a mental illness or mental disorder in 75% (n = 36) of states offering this credential. Only one state did not specify that personal experience with mental health recovery was required to be a peer mental health provider [73]. Family and youth peer providers for child and adolescent mental health were covered in only one publication [67]. Of the 31 states offering a family peer credential, 24 (77%) specified that the person must have lived experience as a parent, caregiver, or other close family member of a child with significant mental or behavioral challenges. Seven (23%) states specified that family peer providers must have experience navigating mental and behavioral treatment systems on behalf of a child. Youth providers were generally considered to have lived experience if they were diagnosed with a mental illness or substance use disorder while under the age of 18 and received treatment services for their mental and/or behavioral conditions during this time. In most cases, eligibility requirements related to lived experience did not require verification, but some states did have applicants sign a statement confirming that they were in recovery.

**Table 2:** Licensing and Credentialing of Peer Providers.

State	Substance Use Peer Provider Title	Substance Use Peer Provider Requirements	Mental Health Peer Provider Title	Mental Health Peer Provider Requirements	Family Peer Provider Title	Family Peer Provider Requirements	Youth Peer Provider Title	Youth Peer Provider Requirements
Alabama	Certified Recovery Peer Support Specialist/ Substance Abuse Peer Support Specialist	Lived experience; HS diploma/GED^4^, state exam; 40 hours training; 16 hours continuing education	Not described	Not described	Mental Health Parent Peer Support Specialist	Lived experience raising a child w/mental illness, behavioral, or emotional disorder; HS diploma/GED*; state-approved training; supervised by a rehabilitative services professional	Certified Mental Health Youth Peer Specialist	Lived experience of having a mental and/r behavioral disorder; HS diploma/GED; state-approved training; supervised by a rehabilitative services professional
Alaska	Peer Recovery Coach*	Not described	Not described*	Not described	Peer Support Specialist**	Lived experience raising a child w/mental illness, behavioral, or emotional disorder; meets qualifications of a behavioral health clinical associate; supervised by a mental health professional clinician	Peer Support Specialist**	Lived experience; meets qualifications of a behavioral health clinical associate; supervised by a mental health professional clinician
Arizona	Peer/ Recovery Support Specialist*	Lived experience; state-approved training	Peer/ Recovery Support Specialist*	Lived experience; state-approved training	Credentialed Parent/Family Support Provider (requirements are the same)	Lived experience raising a child w/mental illness, behavioral, substance use, or emotional disorder; meets requirements to function as a behavioral health professional, behavioral health technician, or behavioral health paraprofessional; competency exam; supervised by a qualified BHT or BHP	*Not offered*	*Not offered*
Arkansas	Peer Recovery Coach*	46 education hours; 500 practice hours; 40 supervision hours; 20 CEUs	Not described*	Not described	Certified Family Support Partner/Certified Peer Support Specialist (requirements are the same)	Lived experience raising a child w/mental illness, behavioral, or emotional disorder; 40 hours of state-approved training; state-approved annual continued training; work under the supervision of a mental health professional	*Not offered*	*Not offered*
California	Certified Peer Recovery Specialist*	100 education hours; 500 practice hours; 35 supervision ours	Not described*	Not described	*Not offered*	*Not offered*	*Not offered*	*Not offered*
Colorado	Certified Peer and Family Specialist*	Lived experience, HS diploma/GED; 60 education hours; 500 practice hours; 25 supervision hours	Not described*	Not described	Peer Support Specialist**	HS diploma/GED; state-approved training; internet access; desire to assist others in their recovery journey	Peer Support Specialist**	HS diploma/GED; established in recovery for a minimum of one year; attendance of all state-approved training days; internet access; desire to assist others in their recovery journey.
Connecticut	Certified Peer Recovery Specialist	50 education hours; 500 practice hours; 25 supervision hours; 10 CEUs	Not described	Not described	*Not offered*	*Not offered*	*Not offered*	*Not offered*
Delaware	Certified Peer Support Specialist*	Lived experience; HS diploma/GED; state exam; state-approved 9-day training; 3 months’ work/volunteer experience; 20 CEUs	Certified Peer Support Specialist*	Lived experience; HS diploma/GED, state exam; state-approved 9-day training; 3 months’ work/volunteer experience; 20 CEUs	*Not offered*	*Not offered*	*Not offered*	*Not offered*
District of Columbia	Certified Peer Specialist*	80 supervision hours; other requirements not described.	Not described*	Not described	Certified Peer Specialist*	Lived experience raising a child w/mental illness, behavioral, or emotional disorder; over 18 years old; HS diploma/GED; resident of DC; willing to create and follow a wellness recovery plan; an 80-hour field practicum with a DC community-based behavioral health provider; certification exam; sign code of ethics	*Not offered*	*Not offered*
Florida	Certified Recovery Peer Specialist (CRPS); Certified Recovery Support Specialist (CRSS)*	CRPS: lived experience; HS diploma/GED; state exam; 40 hours training; 500 hours work/volunteer experience; 10 CEUs. CRSS: 75 training hours; 1000 practice hours; 24 supervision hours; 10 CEUs	Not described*	Not described	Certified Recovery Peer Specialist*	Lived experience raising a child w/mental illness, behavioral, or emotional disorder; HS diploma/GED; 500 hours of supervised work/volunteer experience within the last 5 years, at least 250 hours specific to peer support to others with similar lived experience; 40 hours of state-training; certified by state board; supervision by certified addiction professional	*Not offered*	*Not offered*
Georgia	Certified Peer Specialist	Lived experience; HS diploma/GED; state exam; 40 hours in-class training; 12 CEUs annually	Certified Peer Specialist	Lived experience; HS diploma/GED; state exam; 40 hours in-class training; 12 CEUs annually	Certified Peer Specialist*	Lived experience raising a child w/mental illness, behavioral, or emotional disorder; HS diploma/GED; strong reading comprehension and written communication skills; demonstrated experience with leadership, advocacy, or governance; completion 9 days of state-approved training; state exam; supervised by peer support supervisor	Certified Peer Specialist*	Lived experience; HS diploma/GED; well-grounded in recovery; strong reading comprehension and written communication skills; demonstrated experience with leadership, advocacy, or governance; 9 days of state-approved training; state exam; supervised by peer support supervisor
Hawaii	*Not offered*	*Not offered*	Hawaii Certified Peer Specialist	Not described	*Not offered*	*Not offered*	*Not offered*	*Not offered*
Idaho	Certified Peer Recovery Coach/Certified Recovery Coach (requirements are the same)	Lived experience; HS diploma/GED; 40 hours training; 200 hours work/volunteer experience; 25 supervision hours; 10 hours CEUs annually with 1 hour ethics training	Not described	Not described	Certified Family Support Partner	Lived experience raising a child w/mental illness, behavioral, or emotional disorder; HS diploma/GED; 40 state-approved training hours; post-training exam; 100 hours work experience (with relevant college degree) or 200 hours work exp (without relevant college degree) within a year of completing training; 20 supervision hours; over 18 years old; working knowledge of field	*Not offered*	*Not offered*
Illinois	Certified Recovery Support Specialist; Certified Peer Recovery Specialist	CRSS: 100 hours training, 2000 hours practice, 100 hours supervision, 40 CEUs. CPRS: 100 hours training, 2000 hours practice, 100 hours supervision, 30 CEUs	Not described	Not described	*Not offered*	*Not offered*	*Not offered*	*Not offered*
Indiana	Certified Addiction Peer Recovery Coach I*	Coach I: Lived experience; HS diploma/GED; state exam; 30 hours training; 40 CEUS	Not described*	Not described	*Not offered*	*Not offered*	*Not offered*	*Not offered*
Iowa	Certified Peer Recovery Specialist*	CPRS: lived experience; HS diploma/GED; 46 hours training; 500 hours practice; 25 hours supervision; 20 CEUS	Not described*	Not described	Parent Peer Support Specialist	HS diploma/GED; at least 21 years of age; state-approved training; organization must be accredited	*Not offered*	*Not offered*
Kansas	Certified Peer Mentor*	Lived experience; HS diploma/GED; 15 hours training; CEUs required	Certified Peer Mentor*	Lived experience; HS diploma/GED; 15 hours training; CEUs required	Parent Peer Support Specialist	HS diploma/GED; at least 21 years of age; state-approved training; background check and abuse registry check; associated with a Community Mental Health Center	*Not offered*	*Not offered*
Kentucky	Registered Alcohol and Drug Peer Support Specialist*	60 training hours; 500 practice hours; 25 supervision hours; 10 CEUs	Adult Peer Support Specialist*	Lived experience; HS diploma/GED; state exam; 30 hours training; 6 hours CEU	Family Peer Support Specialist	Self-identified parent or other family member who has lived experience with a child client who has received services for mental health, substance use, or both from at least one child-serving agency; at least 18 years old; HS diploma/GED; 45 hours of state-approved training; 6 annual hours of CEUs	Youth Peer Support Specialist	Lived experience receiving a state-funded service related to emotional, social, behavioral, or substance abuse disability as a child; 18–35 years old; HS diploma/GED; 45 hours of state-approved training; short essay reflecting on lived experience; experience with leadership and advocacy in behavioral health; demonstrate efforts at self-directed leadership development; supervised by a professional
Louisiana	Peer Support Specialist*	Lived experience; HS diploma/GED; state exam; 76 hours training; 10 CEUs	Not described*	Not described	Parent Support Specialist	HS diploma/GED; at least 21; 2 years living or working with a child with serious emotional disturbance OR education in the human services field; certification and 76-hour state approved training; 10 CEUs annually; criminal and professional background checks and motor vehicle screens; supervised by Family Support supervisor	Youth Support Specialist	Lived experience with a behavioral health diagnosis; HS diploma/GED; at least 18 years of age; 76-hour training; background checks; at least 12 months of continuous demonstrated recovery
Maine	Certified International Peer Support Specialist	72 hours practice; other requirements not described	Not described	Not described	Family Support Specialist	Lived experience with a family member who is receiving or has received services and supports related to the diagnosis of a mental illness; state-approved training and receipt of certification	Youth Support Specialist	Lived experience; complete 8 days of training and certification
Maryland	Certified Peer Recovery Specialist*	46 training hours, 500 practice hours, 25 supervision hours, 20 CEUs	Not described*	Not described	Family Peer Support Partner	Lived experience as a caregiver of a child with behavioral health challenges OR experience with state or local services and systems as a consumer who has or had behavioral health challenges; employed by a Family Services Organization; at least 18 years old; supervised by an individual who is 21 or older and has at least 3 years of experience providing family peer-to-peer support or working with children with serious behavioral health challenges and their families;; state-approved training and receipt of certification	*Not offered*	*Not offered*
Massachusetts	Recovery Coach	Lived experience; HS diploma/GED; other requirements in development	Not described	Not described	Family Partner	Lived experience as a caregiver of a youth with special needs; bachelor’s degree in relevant field and one year of experience working with target population OR associate’s degree in relevant field and one year of experience working with children/adolescents/transition age youth OR HS diploma/GED and a minimum of 2 years’ experience working with youth; experience in navigating any of the child- and family-serving systems and in helping others navigate; possess a valid driver’s license, car, and insurance; complete an approved training course	*Not offered*	*Not offered*
Michigan	Certified Peer Recovery Mentor	Lived experience; HS diploma/GED; state exam; 46 training hours; 500 practice hours; 25 supervision hours; 20 CEUs	Not described	Not described	Parent Support Partner	Lived experience as a parent/caregiver of a child with behavioral and mental health needs, and/or intellectual/developmental disability; employed by a Prepaid Inpatient Health Plan or its contract providers; state-approved training	Youth Peer Support Specialist	Lived experience of receiving mental health services as a youth; 18–26 years old; willing to use lived experience in helping others; employed by Prepaid Inpatient Health Pan/Community Mental Health Services Program or its contract providers; state-approved training; regular supervision by a child mental health professional and active member of the treatment team
Minnesota	Certified Peer Recovery Specialist	Lived experience; HS diploma/GED; International Certification & Reciprocity Consortium exam; 46 hours training; 500 practice hours; 25 supervision hours; 20 CEUs	Not described	Not described	Certified Family Peer specialist	Lived experience raising a child with mental illness and navigating the children’s mental health system; employed by a mental health community provider enrolled in Minnesota Health Care Programs; at least 21 years old; HS diploma/GED; demonstrated leadership and advocacy skills; dedication to family-driven and -focused services; state-approved training; certification exam; ongoing supervision from another parent support provider; recertification/renewal every 2 years	*Not offered*	*Not offered*
Mississippi	Certified Peer Support Specialist*	Lived experience; HS diploma/GED; state exam; 34-day training; 250 practice hours; 20 CEUs	Certified Peer Support Specialist*	Lived experience; HS diploma/GED, state exam; 34-day training; 250 practice hours; 20 CEUs	Certified Peer Support Specialist*	Lived experience as family member and/or current or former recipient of mental health and/or substance use services; state resident; employed in state certified program under supervision of mental health professional; HS diploma/GED; minimum of 250 hours of paid work/volunteer hours/activities in a support or advisory role with target population OR a year of related college/educational experience within the last 3 years; two references; state-approved training; state exam	Certified Peer Support Specialist*	Lived experience as a recipient of mental health and/or substance use services; state resident; employed in state certified program under supervision of mental health professional; HS diploma/GED OR at least 16 and enrolled in school/GED program; minimum of 250 hours of paid work/volunteer hours/activities in a support or advisory role with target population or a year of related college/educational experience within the last 3 years; two references; state-approved training; state exam
Missouri	Certified Reciprocal Peer Recovery (CRPR); Certified Peer Specialist (CPS)*	CRPR: Lived experience, HS diploma/GED; International Certification & Reciprocity consortium exam; 500 hours work/volunteer experience; 46 hours training; 25 hours of supervision. CPS: Online exam; 35-hour training; 20 CEUs	Not described*	Not described	Family Support Provider	Lived experience as a family member of a child/youth who had or currently has a behavioral/emotional disorder, or a substance use disorder; HS diploma/GED; state-approved training; supervised by a qualified professional	*Not offered*	*Not offered*
Montana	Certified Behavioral Health Peer Support Specialist*	Lived experience; 40 hours state-approved training; 20 CEUs	Not described*	Not described	*Not offered*	*Not offered*	*Not offered*	*Not offered*
Nebraska	Certified Peer Support Providers*	Lived experience; HS diploma/GED; International Certification & Reciprocity Consortium exam; 60 hours training; 2 years work/volunteer experience; CEUs required	Certified Peer Support Providers*	Lived experience; HS diploma/GED; International Certification & Reciprocity Consortium exam; 60 hours training; 2 years work/volunteer experience; CEUs required	Peer Support Provider*	Lived experience as a parent to a child with a mental health/substance use disorder; at least 19 years old; HS diploma/GED; 2 years relevant paid or volunteer experience; state and/or national certification;^5^ supervised by a professional practitioner	*Not offered*	*Not offered*
Nevada	Certified Peer Support Specialist*	46 hours training; 500 practice hours; 25 supervision hours; 20 CEUs	Certified Peer Support Specialist*	46 hours training; 500 practice hours; 25 supervision hours; 20 CEUs	*Not offered*	*Not offered*	Peer Supporter	Lived experience currently or previously diagnosed with a mental and/or behavioral health disorder; skills & motivation to work with and under supervision by a qualified mental health professional; 475 hours of volunteer or paid work experience; HS diploma/GED; state training; 25 hours of supervised experience; state exam; adherence to code of ethics; must live or work in Nevada at least 51% of the time
New Hampshire	Certified Recovery Support Worker	Lived experience; HS diploma/GED; International Certification & Reciprocity Consortium exam; 46 hours training; 500 hours work experience; 25 hours of supervision; 12 CEUs every 2 years	*Not offered*	Not offered	Family Peer Support	Lived experience as the parent or primary caregiver of a child or youth with emotional or behavioral challenges, who has received supports through the public child- and family-serving systems HS diploma/GED; state driver’s license and/or access to transportation; training	Youth Peer Support	Lived experience of a child/youth with emotional or behavioral health challenges who has received supports through the public child- and family-serving systems; state driver’s license and/or access to transportation; HS diploma/GED; orientation training; pre-service training
New Jersey	Not described	Not described	*Not offered*	*Not offered*	*Not offered*	*Not offered*	*Not offered*	*Not offered*
New Mexico	Certified Peer Support Worker*	lived experience; HS diploma/GED; state exam; 5-day training; 40 hours experience; 40 CEUs every 2 years.	Certified Peer Support Worker*	lived experience; HS diploma/GED; state exam; 5-day training; 40 hours experience; 40 CEUs every 2 years.	Certified Family Support Worker	Lived experience navigating any of the child-/family-serving systems and/or advocating for family members who are involved in the child/family behavioral health systems, as well as an understanding of how these systems operate in New Mexico; at least 18 years old; HS diploma/GED; 40-hour state-approved training program; valid New Mexico address and driver’s license; the ability to manage their own well-being; certification	Certified Peer Support Worker*	Lived experience as a current or former consumer of mental health and/or substance abuse services; at least 18; HS diploma/GED; 40-hour training program; at least two years of mental health or substance abuse recovery; certification
New York	Certified Recovery Peer Advocate	HS diploma/GED; International Certification & Reciprocity Consortium exam; 46 hours training; 1000 hours of experience; 25 supervision hours; 30 CEUs every 3 years	Not described	Not described	Certified Recovery Peer Advocate-Family	Lived experience as a primary caregiver of a youth who has participated in or navigated the addiction services system; at least 18 years old; HS diploma/GED; minimum of 46 hours content-specific training; 500 hours of related work or volunteer experience; at least 25 hours of supervision in a peer role; state exam or another exam by a OASAS^6^-designated certifying body; minimum of 20 hours in the area of family support; 10 hours of CEU per year; supervision by qualified and certified professional	Youth Peer Advocate	Between 18 and 30 years old; self-identify as a person who has first-hand experience with social, emotional, medical, developmental, substance use, and/or behavioral challenges; HS diploma/GED; completes Youth Peer Advocate Training
North Carolina	Certified Peer Support Specialist*	Lived experience; HS diploma/GED; 60 hours training; 20 hours CEUs every 2 years	Not described*	Not described	*Not offered*	*Not offered*	*Not offered*	*Not offered*
North Dakota	Certified Peer Specialist*	100 practice hours; other requirements not specified	Not described*	Not described	*Not offered*	*Not offered*	*Not offered*	*Not offered*
Ohio	Certified Peer Recovery Supporter*	Lived experience; HS diploma/GED; state exam; 56 hours training; 30 CEUs every 3 years	Not described*	Not described	*Not offered*	*Not offered*	*Not offered*	*Not offered*
Oklahoma	Peer Recovery Support Specialist*	Lived experience; HS diploma/GED; state exam; 40-hour training; 12 CEUs annually	Not described*	Not described	Family Support and Training Provider	Lived experience as a family member of a child or youth with serious emotional disturbance OR a minimum of 2 years of experience working with children with serious emotional disturbance OR be equivalently qualified by relevant education or a combination of work experience and education; HS diploma/GED; at least 21 years old; state-approved training; background check; supervised by a Licensed Behavioral Health Professional	*Not offered*	*Not offered*
Oregon	Peer Wellness Specialist (PWS); Peer Support Specialist (PSS)*	PWS: Lived experience; 80 hours training; additional oral health training; 20-hour CEUS annually. PSP: Lived experience; 40 hours training; additional oral health training; 20 CEUs annually	Peer Wellness Specialist; Peer Support Specialist*	PWS: Lived experience; 80 hours training; additional oral health training; 20-hour CEUS annually. PSP: Lived experience; 40 hours training; additional oral health training; 20 CEUs annually	Peer Support Specialist/Peer Wellness Specialist*	Lived experience as a family member of an individual who’s a current or former recipient of addiction or mental health services; at least 18 years old; not on Medicaid provider exclusion list; 40 hours of state training program; criminal background check	Peer Support Specialist/Peer Wellness Specialist*	Lived experience as a recipient of mental health services and/or in at least 2 years of addiction recovery; at least 18 years old; not on Medicaid provider exclusion list; 40 hours of state training program; criminal background check
Pennsylvania	Certified Recovery Specialist (CRS)	Lived experience; HS diploma/GED; state exam; 54 hours training; 3 CEUs every 2 years	Not described	Not described	Certified Family Recovery Specialist	Lived experience, HS diploma, 60 hours training, 30 CEUs	Certified Peer Specialist	Lived experience of receiving mental health services for a serious emotional disturbance or serious mental illness; 18 years of age or older; HS diploma/GED; DHS-approved training; within the last 3 years, 12 months of successful work or volunteer experience, or earn at least 24 credit hours at a postsecondary educational institute; supervised by qualified professional
Rhode Island	Peer Recovery Specialist*	Lived experience; HS diploma/GED; International Certification & Reciprocity Consortium exam; 46 hours training; 500 hours work/volunteer experience; 25 supervision hours; 20 CEUs every 2 years	Not described*	Not described	*Not offered*	*Not offered*	*Not offered*	*Not offered*
South Carolina	Certified Peer Support Specialist*	Lived experience; HS diploma/GED; state exam; 40 hours training; 100 hours work/volunteer experience; 20 CEUs	Not described*	Not described	*Not offered*	*Not offered*	*Not offered*	*Not offered*
South Dakota	*Not offered*	*Not offered*	*Not offered*	*Not offered*	*Not offered*	*Not offered*	*Not offered*	*Not offered*
Tennessee	Certified Peer Recovery Specialist*	Lived experience; HS diploma/GED; 40 hours training; 75 practice hours; 3 supervision hours; 10 CEUs annually	Not described*	Not described	Family Support Specialist	At least 18 years old; HS diploma/GED; self-identify as being or having been the parent or relative caregiver with legal custody of a child or youth with a mental, emotional, behavioral, or co-occurring disorder; statement of personal experience; during last 10 years, have actively participated for at least 12 consecutive months in service planning, system navigation, and building resiliency for a child or youth; state-approved training; mastery of competencies; supervision by a mental health professional	*Not offered*	*Not offered*
Texas	Recovery Support Peer Specialist/Certified Peer Mentor/Peer Recovery Coach (requirements are the same)	Lived experience; HS diploma/GED; online self-assessment and orientation; 8–16 hours training; 46 additional hours of recovery support training; 25 supervision hours; 20 CEUs every 2 years	Certified Peer Mentor	Lived experience; HS diploma/GED; online self-assessment and orientation; 8–16 hours training; 46 additional hours of recovery support training; 20 CEUs every 2 years	Family Support Provider	Lived experience of one cumulative year of experience navigating the mental health system as the parent or primary caregiver of a youth receiving mental health community service; HS diploma/GED; pass a background check; direct therapist supervision	*Not offered*	*Not offered*
Utah	Certified Peer Support Specialist*	Lived experience; state exam; 40 hours training; 30 CEUs every 2 years	Certified Peer Support Specialist*	Lived experience, state exam; 40 hours training; 30 CEUs every 2 years	*Not offered*	*Not offered*	*Not offered*	*Not offered*
Vermont	Not described	Not described	*Not offered*	*Not offered*	*Not offered*	*Not offered*	*Not offered*	*Not offered*
Virginia	Certified Peer Recovery Specialist*	Lived experience; HS diploma/GED; national exam; 72 hours training; 500 hours volunteer/work experience; 25 supervision hours 20 CEUs every 2 years	Not described*	Not described	Family Support Partner	Lived experience; certification by state Department of Behavioral Health and Developmental Services; supervised by a qualified/licensed professional	*Not offered*	*Not offered*
Washington	Certified Peer Counselor*	Lived experience; HS diploma/GED; state exam; online course; 50 hours of training	Certified Peer Counselor*	Lived experience; HS diploma/GED; state exam; online course, 50 hours of training	Certified Peer Counselor - Family and Youth**	At least 18 years old; HS diploma/GED; has been in mental health recovery for at least one year; demonstrates qualities of leadership; reading comprehension and writing skills; willing to share personal story of recovery; online course and application; 40-hour state approved training, passes exam	Certified Peer Counselor - Family and Youth**	At least 18 years old; HS diploma/GED; has been in mental health recovery for at least one year; demonstrates qualities of leadership; demonstrates proficiency in reading comprehension and writing skills; is willing to share their personal story of recovery; online course and application; 40-hour state-approved training; passes exam
West Virginia	Peer Recovery Specialist*	Lived experience; HS diploma/GED; national and state exam; 46 hours training; 500 hours practice; 30 CEUs every 2 years	Not described*	Not described	*Not offered*	*Not offered*	*Not offered*	*Not offered*
Wisconsin	Certified Peer Specialist*	Lived experience; state exam; 6–7-day training; 20 CEUs every 2 years	Certified Peer Specialist*	Lived experience; state exam; 6–7-day training; 20 CEUs every 2 years	Certified Parent Peer Specialist	*Not offered*	*Not offered*	*Not offered*
Wyoming	Certified Peer Specialist*	Lived experience; HS diploma/GED; 32 training hours; 14 CEUs	Certified Peer Specialist*	Lived experience; HS diploma/GED; 32 training hours; 14 CEUs	Family Support Partner	Lived experience as a parent or caregiver of a child with behavioral health needs OR someone with 2 years’ experience working closely with children with serious emotional/behavioral challenges and their families; HS diploma/GED; minimum of 2 years’ experience in a behavioral health setting as a parent, client, or family advocate; at least 21 years old; driver’s license, auto insurance, and transportation; CPR and first aid certification; state-approved training and certification; enrollment as a Wyoming Medicaid Provider; background screenings; participate in ongoing wraparound fidelity monitoring	Youth Support Partner	Lived experience overcoming various systems or obstacles related to mental and behavioral health challenges; HS diploma/GED; 18–26 years old; driver’s license, auto insurance, and transportation; CPR and first aid certification; all required trainings; enrolled as a Wyoming Medicaid Provider; background screenings; participation in ongoing wraparound fidelity monitoring

* Denotes that the credential is the same for peer supporters in multiple domains. In states that offer two integrated credentials ** is used for the second.

^4^High school diploma or General Educational Development equivalent.

^5^Such as the National Certified Peer Recovery Support Specialist (NCPRSS) provided by The Association for Addiction Professionals.

^6^Office of Addiction Services and Supports.

In total, there were 91 unique credentials in 49 states and Washington, D.C. across the four peer service areas (adult substance use, adult mental health, parent or caregiver of a child with mental health problems, and youth mental health). The names of these credentials varied by state and service domain.

Based on the data available, only two states (Mississippi and Oregon) offered a single integrated peer support credential across all behavioral health fields (e.g., the process and requirements for becoming a peer support specialist were the same). Three states (District of Columbia, Florida, and Nebraska) offered an integrated credential for peers in the areas of adult mental health, adult substance use, and family peer support, but not youth peer support; New Mexico offered a credential that encompassed lived experience of mental health, substance use and youth mental health but had a separate credential for family peer support specialists. Over two-thirds of states (68%, n = 34) offered an integrated credential for adult substance use and adult mental health. Fewer states (26%, n = 13) offered separate credentials for peer specialists in both adult mental health and substance use fields. One state (Hawaii) only offered a credential for peers for adult mental health, while three others (New Hampshire, New Jersey, and Vermont) only offered credentials for peers who provided support for substance use. Four states offered an integrated family and youth support credential; 26 states (51%) offered a unique family support peer credential; and 10 states (20%) had unique credentials for youth peer support specialists.

The requirements for peer credentials varied from state to state and across behavioral health fields, though there were common elements. Education, training and supervision requirements were the most common. The majority of credentials explicitly required a high school diploma or equivalent (n = 62, 68%). Training was a universal requirement for peers, but the number of educational hours varied by both state and behavioral health field. For peers supporting adults with mental health or substance use problems, training requirements ranged from 20 hours to 100 hours, with over 50% of certifications requiring between 40 and 48 hours of training [73]. For example, 76 hours of training were required to become a *Peer Support Specialist* for adult substance use in Louisiana, while the *Certified Peer Specialist* for adult substance use in Wyoming only required 32 training hours. Ongoing supervision by either a licensed professional or a peer supervisor was also a universal requirement for all described peer credentials. 41 credentials (45%) across 22 states also required supervised work or volunteer hours as a prerequisite for certification. The number of hours required ranged from fewer than 200 to 1000. Finally, most state credentials (70%, n = 43) also required that peers pass a state or internationally standardized examination.

The minimum age requirement was 18 for nearly all credentials, however, some family support credentials had a 21-year-old minimum age (e.g., *Parent Peer Support Specialist* in Iowa, *Parent Peer Support Specialist* in Kansas, and *Parent Support Specialist* in Louisiana; n = 6). Four states also indicated an age maximum to qualify for their youth peer credential. In Kentucky, for example, individuals must be between the ages of 18 and 35. In Michigan, the age ranged from 18 to 26 years old.

Less common requirements for credentialing included reference letters (in Mississippi); access to transportation (n = 4 credentials); and participation in ongoing fidelity monitoring (n = 2 credentials, both in Wyoming).

### Types of peer support services

Eight of the ten publications included information on types of peer support services that were Medicaid-reimbursable for the fields of adult mental health, adult substance use and child and adolescent mental health. We identified eleven different service types. Psychosocial support was the most frequently mentioned peer service type, reported in all eight publications that discussed this topic (100%). Activities in this category aimed at improving the service recipient’s emotional, mental and social well-being, including building relationships, encouraging resilience, developing coping and problem-solving strategies, and supporting people in stressful situations.

The second most common service type was resource or systems navigation, identified in six of the eight publications (75%) that described service types. This involved helping people to understand and access services within the behavioral health and broader service system. This included activities such as care coordination, assisting individuals in the transition from institutional/residential care back into the community, offering education on how to obtain the best available treatments, and connecting peers to resources.

Recovery and rehabilitative support services were the third most common service type, identified in five publications (63%) and were specific to supporting peers in their recovery process from substance use. Examples of activities within this category included modeling recovery behavior and acting as a recovery coach.

A range of other service types were identified. Health education and health promotion, practical assistance, goal setting and screening and assessments were each identified in half of publications (n = 4). Three publications (38%) identified mentorship as a peer support service type, though no specific definitions were included. Only two publications (29%) identified crisis assistance, advocacy/promoting self-advocacy and data collection and evaluation. Finally, only one publication described counseling or therapy as a type of service delivered by peers, and that was in the area of substance use treatment and care [69].

### Medicaid coverage for peer support services

Eight of the ten publications (80%) provided information about Medicaid coverage for peer support services across 50 states and the District of Columbia. Three states (6%) did not use Medicaid dollars to fund any peer-provided services: South Dakota, Vermont, and Wisconsin. The remaining 48 states used Medicaid funding for peer-support services in at least one of the three service domains of adult substance use, adult mental health services, and child and adolescent mental health. As shown in Table 3, Medicaid dollars were used to fund peer services in adult substance use in 43 states (84%); adult mental health in 43 states (84%); family services in 31 states (61%); and youth behavioral health in 17 states (31%). Only 13 states (25%) had Medicaid reimbursement for peer services in all four domains.

**Table 3:** Medicaid Coverage for Peer Services.

	*Domains of lived experience*				
State	Adult substance use	Adult mental health	Family or caregiver	Youth mental health	Types of Funding
Alabama	x	x	x	x	State Plan, 1115 Waiver
Alaska	x	x	x	x	State Plan, 1115 Waiver
Arizona	x	x	x		State Plan
Arkansas	x	x	x		State Plan
California	x	x			State Plan, 1915(c), 1115 Waiver
Colorado	x	x	x	x	1915(b)
Connecticut		x			State Plan, 1915(c)
Delaware	x	x			1115 Waiver
District of Columbia		x	x		State Plan
Florida	x	x	x		State Plan, 1115 Waiver
Georgia	x	x	x	x	State Plan
Hawaii		x			1115 Waiver
Idaho	x	x	x		State Plan, 1915(b)
Illinois	x	x			State Plan, 1115 Waiver
Indiana	x	x			State Plan
Iowa	x	x	x		1915(b)
Kansas	x	x	x		State Plan and 1915(c)
Kentucky	x	x	x	x	State Plan
Louisiana	x	x	x	x	State Plan, 1915(c)
Maine		x	x	x	State Plan
Maryland	x		x		State Plan
Massachusetts	x	x	x		State Plan, 1115 Waiver
Michigan	x	x	x	x	State Plan, 1115 Waiver, 1915(c), EPSDT
Minnesota	x	x	x		State Plan, 1115 Waiver
Mississippi	x	x	x	x	State Plan
Missouri	x	x	x		State Plan
Montana	x	x			State Plan
Nebraska	x	x	x		State Plan
Nevada	x	x		x	State Plan
New Hampshire	x		x	x	State Plan
New Jersey	x				1115 Waiver
New Mexico	x	x	x	x	State Plan
New York	x	x	x	x	State Plan, 1115 Waiver
North Carolina	x	x			State Plan
North Dakota	x	x			State Plan
Ohio	x				State Plan
Oklahoma	x	x	x		State Plan
Oregon	x	x	x	x	State Plan, 1115 Waiver
Pennsylvania		x		x	State Plan
Rhode Island	x	x			State Plan
South Carolina	x	x			State Plan
South Dakota					N/A
Tennessee	x	x	x		State Plan
Texas	x	x	x		State Plan, 1915(c)
Utah	x	x			State Plan
Vermont					N/A
Virginia	x	x	x		State Plan, 1115 Waiver
Washington	x	x	x	x	State Plan
West Virginia	x				1115 Waiver
Wisconsin					N/A
Wyoming	x	x	x	x	State Plan, 1915(c), 1915(b)

### Type of fundings for peer support services

Four different types of funding were used across states (see Table 4). A Medicaid Waiver (State Plan Amendment, (Authorizing statute: Social Security Act (SSA) 1905(a)(13) and 1915(i)) was the most common, utilized to fund peer services in 42 (82%) states. Most state plan amendments covered rehabilitative services and home- and community-based services, which allowed a state to cover comprehensive peer-support services under the plan. Four states (8%), Colorado, Idaho, Iowa, and Wyoming, utilized non-Medicaid service waivers (Authorizing statute: SSA 1915(b)) which permit states to use the extra savings achieved by providing cost effective care through a Medicaid-managed care program to fund peer-supported services. The third type of funding is a home- and community-based services waiver (Authorizing statute: SSA 1915(c)), which was utilized by seven states (14%). Medicaid demonstration programs (Authorizing statute: 1115 Waiver) were used as a funding sources by 16 states (31%). This funding allows the Secretary of Health and Human Services to waive certain federal Medicaid requirements, and permits programs like peer-supported services that are likely to assist in promoting Medicaid objectives. One state (Michigan) covered youth peer support services under the federally mandated Early Periodic Screening, Diagnostic, and Treatment (EPSDT) benefit that requires states to provide access to any medically necessary Medicaid-coverable services regardless of whether they are covered in the state plan [74].

**Table 4:** Medicaid funding types.

Authorizing statute	Title	Description
Social Security Act (SSA) 1905(a)(13) and 1915(i)	State Plan	*Rehabilitative services*: Allows a state to cover, under its state plan, medical or remedial services recommended by a physician or other licensed health care provider, to reduce physical or mental disability, and restore a Medicaid beneficiary to the best possible functional level. Home- and community-based services: Allows a state to offer a comprehensive package of home- and community- based services under its state plan.
SSA 1915(b)	Non-Medicaid services waiver	Allows a state to use savings it achieves by providing cost effective care through a Medicaid managed care program to furnish additional services to beneficiaries over and above those in its state plan
SSA 1915(c)	Home- and community-based services waiver	Allows a state to provide a broad range of home- and community-based services to beneficiaries who would otherwise require services in an institutional setting, such as a nursing facility.
1115 Waiver	Medicaid demonstration	Allows the Secretary of Health and Human Services to waive certain federal Medicaid requirements and allow costs that would not otherwise be eligible for federal funds for experimental, pilot, or demonstration projects that, in the Secretary’s judgment, are likely to assist in promoting Medicaid objectives.

Some funding mechanisms were more commonly used to support certain domains of peer support than others. For example, five states (Kansas, Louisiana, Michigan, Texas, and Wyoming) utilized a home-and-community-based services waiver to cover family and/or youth peer support services, while funding peer support for adult mental health and adult substance use through other mechanisms such as a state plan amendment.

### Billing codes

Six out of ten publications identified specific billing codes utilized for services delivered by peers, encompassing a variety of responsibilities. The code most commonly used for peer recovery support was H0038, requiring the peer provider to share the direct experience of addiction and recovery. Other billing codes used for peer services included skills training and development (H2014), case management (T1016), parent support and training (S5110), and mental health services not otherwise specified (H0046). These billing codes varied based on in-person vs telephonic, and group vs individual settings, but did not include setting of care stipulations such as clinic or in-home. There was a wide range of cost reimbursement per service and between services billed for peer services across states. There was a median reimbursement rate of $12.33 for code H0038 for peer support services, ranging from $5.75 to $24.49 per 15-minute billing interval [64]. Other services were often reimbursed for lower amounts than H0038, such as individual health education code (i.e., H0036, H2014, H2027, etc.), which had reimbursement rates as low as $3.31 per every 15 minutes.

## Discussion

The purpose of this research was to examine peer services delivered in the fields of adult mental health, adult substance use, and child and adolescent mental health and the role of Medicaid funding for these services. The findings indicated that most states provided Medicaid reimbursement for peer support in some fields but not others, and for certified peer specialists with different domains of lived experience. For example, Delaware provided Medicaid reimbursement for peer specialists in adult substance use and adult mental health, but not for child and adolescent mental health. Alabama reimbursed for all four types of peer support, utilizing both the state plan and 1115 waivers. Overall, more states funded peer services for adult substance use and mental health compared to children and adolescent mental health. For example, in California and South Carolina – states on opposite coasts with very different Medicaid policies – peer services were only eligible for Medicaid reimbursement for adult substance use and mental health, not for family or youth support. The findings also indicated common elements of peer definitions, credentialing processes and types of peer services currently reimbursable by Medicaid.

A primary motivation for conducting this analysis was to inform potential implementation and expansion of peer services for refugee and newcomer communities, given their presumptive Medicaid eligibility [75]. As of 2021, most states have elected to expand Medicaid coverage to qualified immigrants including asylees, refugees, and other newcomers [76]. The findings highlighted opportunities for integrating peer services for refugee and newcomer communities into existing definitions and credentialing processes. The diverse peer service types that are currently reimbursed by Medicaid are relevant to the needs of newcomer communities. The findings also highlighted challenges; especially low reimbursement rates, which will serve as a barrier to integrating people with lived experience of forced displacement into peer service roles. Below, these opportunities and challenges are considered in greater detail along with areas for additional research.

The definitions of peer providers revealed commonalities including lived experience with recovery or healing from a mental health or substance use problem. Peers were also defined by the role that they played, drawing upon their lived experience to provide support, guidance, and assistance to someone currently in those circumstances. Existing definitions of peers also revealed several areas that may need to be adapted when applying the concept to services for refugee and newcomer communities. First, it will be important to expand the conceptualization of lived experiences to included experiences of forced displacement and hardships associated with migration or resettlement. This is particularly important in the context of using peers to deliver prevention-oriented behavioral health services where experiences of a mental health condition or recovery are not as relevant. It will also be important to specify how lived experiences support the mental health and wellbeing of others. Shared cultural and linguistic backgrounds are another dimension of shared life experience relevant for peers working with refugee and newcomer communities. These qualities can promote access to same-language mental health services, enhance culturally relevant care and may reduce the stigma associated with needing and receiving mental health and addiction services [77].

Notably, the language defining eligibility for mental health peer providers does not always reflect the preferences of people with lived experience. Most states require a diagnosis of *mental illness* or *mental disorder,* whereas many people with lived experience prefer the term *mental health condition*, as this carries a less stigmatizing connotation [29]. This is especially relevant for refugee and newcomer communities where stigma is one of the most common barriers to accessing mental health services and thus, may be a barrier to pursuing a peer service delivery credential [77]. It is also unclear whether a mental health diagnosis is a key eligibility criterion for peer refugee services. More relevant might be the experience of forced displacement regardless of a behavioral health diagnosis.

The mosaic of credentials available for peer providers across the nation was vast and complex. In fact, even the term itself was not ubiquitous – states frequently had different terms for adult peers delivering recovery and rehabilitative services for addiction or mental health conditions, family peer supporters who assist the parents of children and youth with behavioral health problems, and youth peers who directly provide support to young people. For example, Kentucky has separate credentials for four types of peer specialists, namely, Registered Alcohol and Drug Peer Support Specialists, Adult Peer Support Specialists, Family Peer Support Specialists, and Youth Peer Support Specialists. Massachusetts used the term *Recovery Coach* for adult substance use peer supporters and *Family Partners* for those providing support to families. This suggests that any peer credential developed or adapted for refugee or newcomer communities may need to be state-specific and aligned with each state’s existing peer credentials and Medicaid eligibility standards.

In addition to the training requirements already in place, it will be important to ensure that training towards credentialing integrates topics particularly germane for refugee and newcomer communities such as principles of trauma-informed care and navigating significant cultural stigma associated with accessing behavioral health services [77]. Furthermore, training for peer providers will also benefit from discussion of boundaries when working within one’s own co-ethnic community, self-disclosure practices, and self-care strategies to mitigate the impact of revisiting prior traumatic or challenging life experiences, particularly for individuals living in ongoing conditions of chronic stress [40]. For example, a peer psychosocial support program for refugees and immigrants in the U.S. integrated training on topics including professional boundaries and cultivation of self-care practices and these were found to be extremely important to peer providers [78].

Future efforts to develop training can draw on work being done in global mental health settings specific to peers and non-specialists [79]. For example, the Ensuring Quality in Psychological Support (EQUIP) is a joint project led by the World Health Organization and United Nations Children’s fund to assess and train non-specialists to deliver mental health and psychosocial support services [80]. They have developed competency tools and training materials in support of this goal. Specific to peer providers, the Global Mental Health Peer Network builds the capacity of people with lived experience to deliver services and advocate for mental health policy and practice [28]. Of course, fully elaborating relevant training topics will ultimately require the input and participation of refugees and newcomers who currently provide peer support, and who are best positioned to speak to the full range of skills and competencies needed to deliver care effectively.

We identified eleven different peer services covered by Medicaid. It is likely that newcomers will benefit from these diverse services including psychosocial support, systems navigation, practical assistance for meeting basic needs, and education about their new communities and resources available. Similar models have been tested for refugees in research studies [81,82]. For example, peer support groups for Bhutanese and Iraqi refugees in the U.S. were found to improve participants’ confidence in accessing resources, sense of independence, and connection with their communities [81]. There may also be other types of services specific to the unique needs of these populations that are yet to be developed, adapted or evaluated for delivery by peers. One example is peer counseling. Although only one publication in this review described counseling as a service delivered by peers, there is a growing evidence base in global mental health settings related to peer and non-specialist delivery of evidence-based individual, group and family therapies [40]. For example, a 2011 Zimbabwe study found that problem-solving therapy delivered by peers resulted in clinically meaningful improvement in symptoms of common mental disorders [83]. Future work in the U.S can draw upon this emerging evidence base and test use of similar models as a further strategy for meeting the mental health needs of newcomers.

Reimbursement rates for peer services varied considerably between states, with even the top end of the peer provider range notably low in comparison to reimbursement rates reported for non-peer service providers [84]. Low rates pose challenges for equitable compensation of peer support services and undervalue the importance of integrating people with lived experience into service systems. This may be especially problematic for refugee and newcomer individuals who may be the sole income-earner within their families. Moreover, refugee and newcomers typically arrive to the U.S. with little to no financial assets, often having to leave their country quickly or exhausting available resources while displaced in refugee camps. When they arrive to the U.S., they need to accumulate financial and other resources rapidly to meet basic needs, making peer pay rates unattractive.

These findings also raise questions about the financial viability and feasibility of relying on Medicaid to fund peer services for refugee and other displaced communities. Refugee service organizations may consider Medicaid as one of several funding sources that they access to cover costs of providing mental health services. Such braided funding approaches are common and reduce organizations’ vulnerability to funding cuts of a single funder or changes in funders’ priorities. It is unknown how the current U.S. political administration will impact refugee resettlement and service delivery over the next few years. Regardless, the number of refugees currently in the U.S. is large and their needs for mental health care are significant. This suggests that our findings will continue to have relevance to the development of refugee social services under the current as well as future administrations.

Building on these findings, we propose several policy recommendations with the potential to better integrate peer mental health services for newcomers into the Medicaid framework. Medicaid is a federal-state program where both levels of government share the cost of providing healthcare to low-income individuals. At the federal and state levels, we recommend that Medicaid coverage be expanded to include prevention-oriented mental health services for refugee and newcomer populations. This is especially important given the considerable size of these populations in the U.S., their well-documented mental health risks, and the substantial benefits associated with delivering services early on before symptoms and problems become severe and intractable. In addition, we recommend that demonstration projects be conducted to determine the best ways to integrate refugee and newcomer peers into states’ current licensing and credentialing structures, and to evaluate the impact of peer-delivered services and supports on mental health and psychosocial outcomes of newcomer populations. States where Medicaid funding is already available for peer services across mental health fields and lived experience domains might be ideal contexts to conduct these demonstration projects. Simultaneously, we recommend that the current landscape of peer services in refugee service organizations be documented. This includes documenting current practices, conceptualizations of peers, barriers and challenges, training and supervision approaches and promising peer service models. These findings can inform future Medicaid funded peer services. One option is for states to fund projects addressing these recommendations via Medicaid waivers (e.g., Authorized by waiver 1115), as these have fewer restrictions than other types of Medicaid waivers in the interest of cultivating innovative health programs. It is also advised that philanthropic and charitable organizations support this work. Finally, policy changes at the state and federal level are needed to remedy low reimbursement rates for peer services which will function as a disincentive for refugee service organizations to rely on Medicaid to support peer service models or to implement them at all. Another recommendation is that both states and employers address the typically low wage rates paid to those in peer positions, since refugee and newcomer populations in particular need a livable wage to achieve an acceptable standard of living and economic integration [85].

Some limitations are important to note. First, as with all reviews, our study is limited by the information available in the selected publications. In some cases, information related to definitions, credentialing and peer service types was limited. We found only one publication focused on the use of family and youth peers in child and adolescent mental health. Additionally, state Medicaid policies are changing, and it is possible that policies related to funding peer services have been updated since this review was conducted and may change in the future. In this review, we focus exclusively on Medicaid-funded peer services. Thus, findings may not pertain to definitions, training and credentialing norms or services funded through other means. Furthermore, while the eligibility requirements for Medicaid vary on a state-by-state basis, it is limited to low-income individuals. Thus, the services examined in this review would not encompass those available to middle- or high-income individuals who have private insurance. Despite these limitations, the current analysis enables a broader picture of the state of Medicaid financed peer services, highlighting areas of opportunity and challenge vis a vis implementation of peer services for refugee and newcomer communities.

## Conclusion

Drawing on evidence domestically and in global settings, peer services have an important role to play in meeting the known and significant mental health needs of refugee and newcomer communities arriving in the U.S. In this analysis, we identified opportunities to build upon existing Medicaid policies and practices and integrate refugee and newcomer peers into existing reimbursement, credentialing processes and service delivery models. We also identified challenges including significant variability in Medicaid funding practices between states, limited funding for peers for child and adolescent mental health and low rates of reimbursement that will compromise the financial sustainability of peer services overall. The lived experience community and peer organizations have advocated for fair and equal remuneration practices for peer expertise, acknowledging its critical role in the sustainability of peer-led service delivery [86]. Translating these findings into genuine workforce and service opportunities will require the participation of displaced persons with lived experience, refugee and newcomer service organizations and policymakers and a shared mission to improve mental health services for refugees and newcomers fleeing war and violence.

## Supporting information

S1 Appendix S1_Appendix_Medicaid_PeerServices. (DOCX)

S1 Prisma S1_PRISMA-ScRChecklist_PeersServices_Medicaid. (PDF)
