# Correction: Peer services in behavioral health: A scoping review of Medicaid funding to inform policy and practice for refugee and newcomer populations in the U.S

**DOI:** 10.1371/journal.pmen.0000480

**Published:** 2025-10-24

**Authors:** Mary Bunn, McKenzie Graunke, Xia Liu, Nihmot Adebayo, Sara Izquierdo, Charlene Sunkel, Judith A. Coo

The fourth author’s name is spelled incorrectly. The correct name is: Nihmot Adebayo. The correct citation is: Bunn M, Graunke M, Liu X, Adebayo N, Izquierdo S, Sunkel C, et al. (2025) Peer services in behavioral health: A scoping review of Medicaid funding to inform policy and practice for refugee and newcomer populations in the U.S. PLOS Ment Health 2(7): e0000359. https://doi.org/10.1371/journal.pmen.0000359
