# Supplementary material for: Peer services in behavioral health: A scoping review of Medicaid funding to inform policy and practice for refugee and newcomer populations in the U.S
Source: PLOS Ment Health. 2025 Jul 10;2(7):e0000359. doi: 10.1371/journal.pmen.0000359 (PMC12798223; doi:10.1371/journal.pmen.0000359)
Supplement: S1 Appendix — (DOCX) [file pmen.0000359.s001.docx]

S1 Appendix: Scoping Review Search Terms

PubMed:

(medicaid[tiab] OR Medicaid[mesh]) AND (peer[tiab] OR peers[tiab] OR peer-to-peer[tiab] OR "Peer Group"[mesh] Not Peer-review[tiab])

PAIS Index&PsychInfo:

(MAINSUBJECT.EXACT.EXPLODE("Medicaid") OR ti,ab(medicaid)) AND (MAINSUBJECT.EXACT.EXPLODE("Peer Groups") OR MAINSUBJECT.EXACT.EXPLODE("Peers") OR ti,ab(peer OR peers OR peer-to-peer Not peer-review))

Web of Science

(Medicaid) and (Peer OR Peers Or Peer-to-Peer Not peer-review)
